# Supplementary material for: Ancient Gene Duplications, Rather Than Polyploidization, Facilitate Diversification of Petal Pigmentation Patterns in Clarkia gracilis (Onagraceae)
Source: Mol Biol Evol. 2021 Aug 16;38(12):5528–38. doi: 10.1093/molbev/msab242 (PMC8662608; doi:10.1093/molbev/msab242)
Supplement: msab242_Supplementary_Data [file msab242_supplementary_data.pdf]

Supplementary Material

Article title: Ancient gene duplications, rather than polyploidization, facilitate diversification of petal pigmentation patterns in *Clarkia gracilis* (Onagraceae)

Authors: Rong-Chien Lin, Mark D. Rausher

Table S1. Primers used in this study.

Table S2. Anthocyanin genes identified from the transcriptomes of *Clarkia gracilis albicaulis*.

Table S3. Synonymous substitution rate (d_S) along the branches leading to *Clarkia gracilis* and to its progenitor species, *C. amoena huntiana* and *C. lassenensis*.

Table S4. Voucher information for the *Clarkia* plants used in this study.

Fig. S1. Alignment of *Clarkia R2R3-MYB* sequences (*A*) *MYB1*, (*B*) *MYB6*, (*C*) *MYB11* and (*D*) *MYB12* from *C. gracilis sonomensis*, *C. g. albicaulis*, *C. amoena huntiana* and *C. lassenensis*.

Fig. S2. Spatiotemporal expression of *MYB1*, *MYB6*, *MYB11* and *MYB12* in the petals of (*A*) *Clarkia amoena huntiana*, (*B*) *C. lassenensis*, (*C*) pink-cupped *C. gracilis sonomensis*, (*D*) white-cupped *C. g. sonomensis*, (*E*) *C. g. albicaulis*.

Fig. S3. Gene tree used to estimate d_S (synonymous substitution rate) along branches.

Methods S1. Transcriptomics

Table S1. Primers used in this study. [†]Primers from Martins *et al*. 2017. [‡]Primers from Lin and Rausher 2021. [a]Semi-qPCR primers used for *Clarkia lassenensis*.

Primers for coding region sequencing

Target species: *Clarkia amoena huntiana*

Primer Name	Primer Sequence (5’-3’)
cMYB1-F[†]	ATGAATAAGGTAGGACTTAGAAAGG
cMYB1-R[†]	TTAAAATATGTCATCAAAATAAAGCTCATCC
cMYB6-3F[‡]	TGCTACAGAAAGTCTAACGT
cMYB6-1R[‡]	ACCGCTGATTTATTTGAAACCCT
cMYB11-5F[‡]	AAAAACCAGAAGAAAACCCA
cMYB11-6R	AATTTCACAGTTTAATCATTTG
cMYB12-9F	AATGAAGGAAGGTCTAAGGAA
cMYB12-5R	CTACAACTGAAATATGTCGTGATC

Target species: *Clarkia lassenensis*

Primer Name	Primer Sequence (5’-3’)
cMYB6-4F	CATGGGTGGTGTTCCTTGGA
cMYB6-5R	TTACAGAGAGTTATTCCACAGATCG
cMYB11-7F	AATGAAGGGAGAATTAAGGAAG
cMYB11-5R	TTACTTGGAAATATGATTCTACACA
cMYB12-11F	ATGCACGCTCTACAATAAAACG
cMYB12-6R	TTTAGTATAACGGCATAATTTCT

Target species: *Clarkia gracilis albicaulis*

Primer Name	Primer Sequence (5’-3’)
cMYB6-3F[‡]	TGCTACAGAAAGTCTAACGT
cMYB6-1R[‡]	ACCGCTGATTTATTTGAAACCCT
cMYB11-3F	GTGAGTCGCATGGCTGT
cMYB11-2R[‡]	CACAGTTTAATCATTTGATTC

Primers for semi-quantitative assessment

Primer Name	Primer Sequence (5’-3’)
cMYB1-gF[†]	TGGTCACTCATAGCAGGAAGA
cMYB1-Q1R	TCATCAACCAGTCCGACCAA

ClMYB1-1R[a]	GGGTTATACCCACTCTCAGTTCC
cMYB6-Q1F[‡]	GACGAACCCACCGAATCAAG
cMYB6-Q1R[‡]	CAGATCGTCCCAGTCCCATT
ClMYB6-Q1F[a]	CGGTGTCGGTCTTGGAGATA
ClMYB6-Q1R[a]	TCATCCCAATCCCATTTTCTGC
cMYB11-Q1F[‡]	GACAAGGAGGTGATGAATTGGT
cMYB11-Q1R[‡]	ATTCTACACATTCATGGAGTCGA
cMYB12-Q1F[‡]	GAGCAAGATTCGGTCAAAGT
cMYB12-1R[‡]	TATTCCGTTACAATGAGGCT
GAPDH-Q1F[‡]	GAGGCATCAGAGACCCACAT
GAPDH-Q1R[‡]	CACGACACGAGCTTCACAAA

Table S2. Anthocyanin genes identified from the transcriptomes of *Clarkia gracilis albicaulis*. The transcriptomes were obtained from pink background and white band of the *C. g. albicaulis* petals, following the protocol described in supplementary methods S1. Gene expression levels were estimated as FPKM values by mapping reads to the transcriptome references of pink background and white band, separately.

Contig ID	Gene	Expression (FPKM)	
		Pink background	White band
Pink background reference			
RCL6_27068_c0_g1	*Chs*	1350.66	1138.03
RCL6_21692_c0_g1	*Chi*	74.56	26.34
RCL6_23311_c0_g4	*F3h*	45.94	50.03
RCL6_27159_c0_g1	*F3'h*	15.98	27.28
RCL6_23784_c0_g1	*F3'5'h*	649.53	381.47
RCL6_23661_c0_g1	*Dfr*	757.58	121.86
RCL6_26489_c1_g1	*Dfr3*	66.31	1140.31
RCL6_26565_c4_g2	*Ans*	628.81	45.37
RCL6_22269_c2_g4	*Uf3gt*	216.18	154.25
RCL6_24035_c1_g1	*MYB6*	858.87	595.14
RCL6_21823_c2_g1	*MYB11*	121	46.94
RCL6_28257_c0_g1	*bHLH1*	39.6	34.93
RCL6_20240_c0_g1	*bHLH2*	4.88	5.04
RCL6_23159_c0_g2	*WDR1*	20.9	14.74
RCL6_21868_c0_g1	*WDR2*	14.88	13.33
White band reference			
RCL7_26363_c0_g2	*Chs*	1372.81	846.27
RCL7_20945_c0_g1	*Chi*	108.08	25.05
RCL7_20216_c0_g4	*F3h*	78.11	70.13
RCL7_26446_c0_g1	*F3'h*	16.34	22.74
RCL7_23161_c0_g1	*F3'5'h*	639.85	290.18
RCL7_28129_c1_g1	*Dfr1*	610.03	77.41
RCL7_20076_c4_g1	*Dfr3*	52.54	705.62
RCL7_23829_c0_g1	*Ans*	579.14	35.97
RCL7_20317_c4_g2	*Uf3gt*	189.3	107.65

RCL7_23760_c0_g1	*MYB6*	664.67	354.33
RCL7_25096_c3_g1	*MYB11*	117.44	36.33
RCL7_27788_c0_g4	*bHLH1*	37.33	25.92
RCL7_20277_c0_g2	*bHLH2*	1.85	1.52
RCL7_22704_c0_g1	*WDR1*	19.48	11.32
RCL7_22679_c0_g1	*WDR2*	10.62	7.38

Table S3. Synonymous substitution rate (d_S) along the branches leading to *Clarkia gracilis* and to its progenitor species, *C. amoena huntiana* and *C. lassenensis*. NA, not applicable.

	Branch(es)		
Clade	*C. gracilis*	*C. amoena huntiana*	*C. lassenensis*
MYB6	0.0371	NA	0.1415
MYB1	0.0218	0.0031	NA
MYB11	0.0248	0.0025	NA
MYB12	0.0085	0.0025	NA

Table S4. Voucher information for the *Clarkia* plants used in this study. RSABG, seeds obtained from Rancho Santa Ana Botanical Garden (Claremont, CA, USA). NFW, seeds from the collection of Norman F. Weeden. TRM, seeds provided by Talline R. Martins (University of Florida, Gainesville, FL, USA). LDG, seeds from the collection of Leslie D. Gottlieb.

Specimen	Voucher	Location	Phenotype
C. amoena huntiana	RSABG 15875	Mendocino County, CA	Central-spotted, pink-cupped
C. amoena huntiana	RSABG 15876	Marin County, CA	Central-spotted, pink-cupped
C. lassenensis	NFW 84b	Shasta County, CA	Basal-spotted, white-banded
C. lassenensis	NFW 152	Shasta County, CA	Basal-spotted, white-banded
C. gracilis sonomensis	TRM 14.6 (LDG 8513)	Sonoma County, CA	Central-spotted, pink-cupped
C. gracilis sonomensis	TRM 14.26 (LDG 8513)	Sonoma County, CA	Central-spotted, white-cupped
C. gracilis albicaulis	TRM 040 (Butte12)	Butte County, CA (Collected by B. Barringer)	Basal-spotted, white-banded

A

```
CgsMYB1  ATGAATAAGGTAGGAGTTAGAAAGGGTGGTTGGACTGCAAATGAAGATGC  50
CgaMYB1  ATGAATAAGGTAGGACTTAGAAAGGGTGGTTGGACTGCAAATGAAGATGC  50
CahMYB1  ATGAATAAGGTAGGACTTAGAAAGGGTGGTTGGACTGCAAATGAAGATGC  50
 ClMYB1  -------------------------GTAGTTGGACAGCAAAAGAAGATGC  25

CgsMYB1  CCTACTCAAGCAATGCGTTCAAACTTATGGAGAAGGCAACTGGCATCTAG  100
CgaMYB1  CCTACTCAAGCAATGCATTCAAACTTATGGAGAAGGCAATTGGCATCTAG  100
CahMYB1  CCTACTCAAGCAATGCGTTCAAACTTATGGAGAAGGCAACTGGCATCTAG  100
 ClMYB1  CCTACTGAAGCAATGCGTTCAAACCTATGGAGAAGGTAACTGGCATCTAG  75

CgsMYB1  TCCCCGATAGAGCAGGTTTGAATAGGTGCAGAAAAAGTTGCCGATTAAGG  150
CgaMYB1  TCCCCGATCGAGCAGGTTTGAATAGGTGCAGAAAAAGTTGCCGATTAAGG  150
CahMYB1  TCCCCGATAGAGCAGGTTTGAATAGGTGCAGAAAAAGTTGCCGATTAAGG  150
 ClMYB1  TCCCCGATAGAGCAGGTTTGAATAGGTGCAGAAAAGGTTGCAGATTGAGG  125

CgsMYB1  TGGCTTAACTATCTGAAACCAGGCTTAAACCGAGAAGAGTTCCAAGAAGA  200
CgaMYB1  TGGCTTAACTATCTGAAACCAGGCTTAAACCGAGAAGAGTTCCAAGAAGA  200
CahMYB1  TGGCTTAACTATCTGAAGCCAGGCTTAAACCGAGAAGAGTTCCAAGAAGA  200
 ClMYB1  TGGCTTAACTATCTAAAGCCAGGCATAAATCGAGGAGAGTTCCAAGAAGA  175

CgsMYB1  TGAAATTGACTTGATTATTAGGCTTCACAAGCTTTTTGGCAATAAATGGT  250
CgaMYB1  TGAAATCGACTTGATTATTAGGCTTCACAAGCTTTTCGGCAAAAAATGGT  250
CahMYB1  TGAAATTGACTTGATTATTAGGCTTCACAAGCTTTTCGGCAAAAAATGGT  250
 ClMYB1  TGAAATTGACTTGATTATTAGGCTTCACAAGCTTTTTGGCAAAAAATGGT  225

CgsMYB1  CACTCATAGCAGGAAGACTTCCTGGAAGAACAAGCAATGATATAAAGAAT  300
CgaMYB1  CACTCATAGCAGGAAGACTTCCTGGAAGAACAAACAATGATATAAAAAAT  300
CahMYB1  CACTCATAGCAGGAAGACTTCCTGGAAGAACAAGCAATGATATAAAGAAT  300
 ClMYB1  CACTCATAGCAGGAAGACTTCCCGGAAGAACAAGCAACGATATAAAGAAT  275

CgsMYB1  TACTGGTACACCCATATTGCCAAGAAATTA---------CCTGCAGAACC  341
CgaMYB1  TACTGGTACACCCATATTGCCAAGAAATTA---------CCTGCAGAACC  341
CahMYB1  TACTGGTACACCCATATTGCCAAGAAATTA---------CATGCAGAACC  341
 ClMYB1  TACTGGAATAGTCATATTGTCAAGAAATTAGCCAAAGCTGCTGCACAACC  325

CgsMYB1  AGTTATATCGCAGGAAAATTCAGTCAAACACCACGCCATAATAAGGCCTA  391
CgaMYB1  AATTATATCGCAGGAAAATTCAGTCAAACACCACGCCATAATAAGGCCTA  391
CahMYB1  AATTATATCGCAGGAAAATTCAGTCAAACACCACGCCATAATAAGGCCTA  391
 ClMYB1  AATTATTTCGCAGGAAAATTCAGTCAAAGAACACGTCATAATAAAACCTA  375

CgsMYB1  TTGCAGGAAGACCGACCAAAGGGATGCAGTTTGGTATGAA----------  431
CgaMYB1  TTGCAGGAAGACCTACCAAAGGCATGCAGTTTGGTATGAA----------  431
CahMYB1  TTGCAGGAAGACCGACCAAAGGGATGCAGTTTGGTATGAA----------  431
 ClMYB1  TTGCAGGAAGACTTACCAAAGGGATGAAGTTTGGCATGAATATCGTCTTA  425

CgsMYB1  --TGTCACCTCTGATCAGCCTCCACCACCTCTGGAGAATTGGTCGGACTG  479
CgaMYB1  --TGTCACCTCTGATCAGCCTCCACCACCTCTGGAGAATTGGTCGGACTG  479
CahMYB1  --TGTCACCTCTGATCAGCCTCCACCACCTCTGGAGAATTGGTCGGACTG  479
 ClMYB1  GGTGCCACCTCTGATCAGCCTCCACCACCTTGGGAGAATTGGTGGGACAG  475

CgsMYB1  GTTGATGATGGACGATGATATTAATTATTATGATAATGGTGGTGGTTGTT  529
CgaMYB1  GTTGATGATGGACGATGATATTAATTATTATGACAATGGTGGTGGTTGTT  529
CahMYB1  GTTGATGATGGACGATGATATTAATTATTATGATAATGGTGGTGGTTGTT  529
 ClMYB1  GTTGATGATGGGTGATGATAATAATTATTATGATTTTGGTGGTGGTTGTT  525
```

Fig. S1. (to be continued on the next page)

```
CgsMYB1  CTGCCTCGGAAGGCCACTGCACCACCGCTGCCAATGGCTGCTACGACATG  579
CgaMYB1  CCGCCTCGGAAGGCCACTGCACCACCGCTGCCAATGGCTGCTACGACATG  579
CahMYB1  CTGCCTCGGAAGGCCACTGCACCACCGCTGCCAATGGCTGCTACGACATG  579
 ClMYB1  CCGCCTCAGGAGGCCACTGCACCACCGCTACCAATG---GCTACCACCTG  572

CgsMYB1  CAGATAGAATCTC---CGTGGGCGGCAATAGTAGGCGGATTCACCGAGGG  626
CgaMYB1  CAGATAGAATCTC---CGTGGGCGGCAATAGGAGGCGGATTCACCGAGGG  626
CahMYB1  CAGATAGAATCTC---CGTGGGCGGCAATAGTAGGCGGATTCACAGAGGG  626
 ClMYB1  CAGATAGGATCTCCGTCGTGTGCGGCAGAAGGA---------ACTGAGAG  613

CgsMYB1  TGGGAATAACCCGGATGAGCTTTATTTTGATGACATATTTTAA         669
CgaMYB1  TGGGAATAACCCGGATGAGCTTTATTTTGATGACATATTTTAA         669
CahMYB1  TGGGAATAACCCGGATGAGCTTTATTTTGATGACATATTTTAA         669
 ClMYB1  TGGGTATAACCC-------------------------------         625
```

Fig. S1. (to be continued on the next page)

B

```
CgsMYB6  ATGGGTGGTGTTCCTTGGACTGAAGAAGAGGATCTTCTGCTTAAGAAATG  50
CgaMYB6  ATGGGTGGTGTTCCTTGGACTGAAGAAGAGGATCTTCTGCTTAAGAAATG  50
CahMYB6  ATGGGTGGTGTTCCTTGGACTGAAGAAGAGGATCTTCTGCTTAAGAAATG  50
 ClMYB6  -------------------CTGAAGAAGAAGATCTTCTGCTTAAGAAATG  31

CgsMYB6  CGTCGAGCAGTTCGGCGAAGGGAAATGGCACCGGGTTCCGCTTTTAGCCG  100
CgaMYB6  CGTCGAGCAGTTCGGCGAAGGGAAATGGCACCGGGTTCCGCTTTTAGCCG  100
CahMYB6  CGTCGAGCAGTTCGGCGAAGGGAAATGGCACCGGGTTCCGCTTTTAGCCG  100
 ClMYB6  CGTCGAGCAGTTCGGTGAAGGGAAATGGCACCGGGTTCCGCTTTTAGCCG  81

CgsMYB6  GTCTGAACAGGTGCAGGAAGAGTTGCAGACTGAGGTGGCTGAATTATCTT  150
CgaMYB6  GTCTAAACAGGTGCAGGAAGAGTTGCAGACTGAGGTGGCTGAATTATCTT  150
CahMYB6  GTCTGAACAGGTGCAGGAAGAGTTGCAGACTGAGGTGGCTGAATTATCTT  150
 ClMYB6  GTCTAAACAGGTGCAGGAAGAGTTGCAGACTGAGGTGGCTGAACTACCTC  131

CgsMYB6  CGACCGAATATCAAGAGAGGGAGCTTTACTCAAGATGAAGTCGAGCTCAT  200
CgaMYB6  CGACCGAATATCAAGAGAGGGAGCTTCACTCAAGATGAAGTCGAGCTCAT  200
CahMYB6  AGACCGAATATCAAGAGAGGGAGCTTCACTCAAGATGAAGTCGAGCTCAT  200
 ClMYB6  CGACCGAATATCAAGAGAGGGAGCTTCACTCAAGATGAAGTCGAGCTCAT  181

CgsMYB6  CATCAAGCTCCATAAGCTTGTCGGGAATCGGTGGTCGATGATTGCCGGAA  250
CgaMYB6  CATCAAGCTCCATAAGCTTGTCGGGAATCGGTGGTCGATGATTGCCGGAA  250
CahMYB6  CATCAAGCTCCATAAGCTTGTCGGGAATCGGTGGTCAATGATTGCCGGAA  250
 ClMYB6  CATCAAGCTCCACAAGCTTGTCGGGAATCGGTGGTCGATGATTGCTGGAA  231

CgsMYB6  GACTCCCGGGAAGAACAGCTAACGATGTCAAGAACTTTTGGAACTGTCAT  300
CgaMYB6  GACTCCCTGGAAGAACAGCTAATGATGTCAAGAACTTCTGGAACTGTCAT  300
CahMYB6  GACTCCCGGGAAGAACAGCTAACGATGTCAAGAACTTTTGGAACTGTCAT  300
 ClMYB6  GACTCCCGGGAAGAACAGCGAACGATGTCAAGAACTTTTGGAACTGCCAT  281

CgsMYB6  CTAAGCAAAAAGCTGACTGCCGAACAAATGAGCATCGACCCTGAACAGAG  350
CgaMYB6  CTAAGCAAAAAGCTGACTGCCGAACAAATGAGCATCGACCCTGAACGAAG  350
CahMYB6  CTAAGCAAAAAGCTGACTGCCGAACAAATGAGCATCGACCCTGAACAGAG  350
 ClMYB6  CTGAGCAAAAAGCTGACTGCCGAACAAATGAGCATCGACCCTGAACGGAG  331

CgsMYB6  AATAGACAACCTAGTCCCGATAATAATGCCCCAACCGCGAAACCCTACAT  400
CgaMYB6  CATAGATAGCCTAGTCCCGGTCATAATGCCACAACCGCGAAACCCTGGAT  400
CahMYB6  AATAGACAACCTAGTCCCGATCATAATGCCCCAACCACGAAACCCTACAT  400
 ClMYB6  CATAGACAGTCTAGTCCCGGTCATAATGGCACAACCGCTAAAATCGGTAT  381

CgsMYB6  CCGTCTCAAGAAAACCGAACAAAAGAGACCAAGAAGTTGGGACTTCTATA  450
CgaMYB6  CCATCTCGAGAAAACCGAACAAAAGAGACCAAGAAGTTGGTACCTCTATA  450
CahMYB6  CCGTCTCGAGAAAACCGAACAAAAGAGACCAAGAAGTTGGGACTTCTATA  450
 ClMYB6  CCGTCTCGAGAAAACTGAACAAAAGAGAGCAAGAAGTTGGGACTTCTATA  431

CgsMYB6  GTGACACTTCCAAGTGTCGGTGAAGATGGAGCAATGAACGCAGTTCAAGT  500
CgaMYB6  GTGACACTTCCAAGTGTCGGTGAAGATGGAGCAATGAACGCAGTTCAAGT  500
CahMYB6  GTGACACTTCCAAGTGTCGGTGAAGATGGAGCAATGAACGCAGTTCAAGT  500
 ClMYB6  GTGACACTTCCAAGCGTCGGTGAAGATGGGGCAATGAATGCAATTCAAGT  481

CgsMYB6  TATCGATGATGGAAAGACGAACCCACCGAATCAAGAACACAGTGTCAGTC  550
CgaMYB6  TATCGATGATGGAAAGACAAACCCACCGAATCAAGAACACGGTGTCGGTC  550
CahMYB6  TATCGATGATGGAAAGACGAACCCACCAAATCAAGAACACGGTGTCAGTC  550
 ClMYB6  TATCGATCATGGAAAGATGAACCCTGCAACTCAAGAACACGGTGTCGGTC  531
```

Fig. S1. (to be continued on the next page)

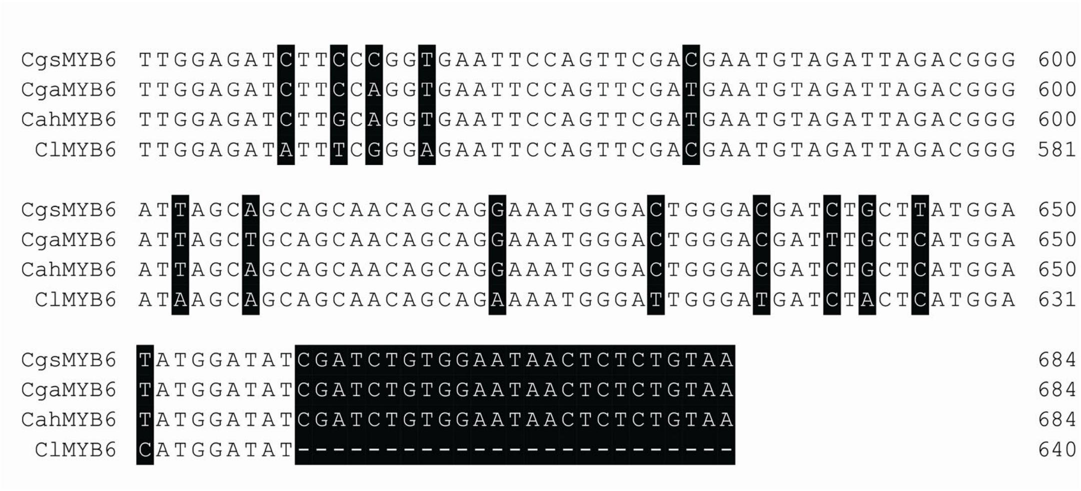

Fig. S1. (to be continued on the next page)

C

```
CgsMYB11 ATGAAGGGAGAATTAAGGAAGGGTGTTTGGAATGCAGAAGAAGATGCTCT 50
CgaMYB11 ATGAAGGGAGAATTAAGGAAGGGTGCTTGGAATGCAGAAGAAGATGCTCT 50
CahMYB11 ATGAAGGGAGAATTAAGGAAGGGTGCTTGGAATGCAGAAGAAGATGCTCT 50
 ClMYB11 ---------------------GGTGCATGGAATGCAGAAGAAGATGCTCT 29

CgsMYB11 CCTCAAGCAATGCATTCAAACTTATGGAGAAGGAAAATGGCATCTTGTTC 100
CgaMYB11 CCTCAAGCAATGCATTCAAACTTATGGAGAAGGAAAATGGCATCTTGTTC 100
CahMYB11 CCTCAAGCAATGCATTCAAACTTATGGAGAAGGAAAATGGCATCTTGTTC 100
 ClMYB11 CCTCAAGCAATGCATTCAAACTTATGGAGAAGGCAAATGGTATCTTGTTC 79

CgsMYB11 CTGCAAGAACAGGACTCAATAGGTGCAGAAAAAGTTGCAGGTTGAGGTGG 150
CgaMYB11 CTACAAGAACAGGACTCAATAGGTGCAGAAAAAGTTGCAGGTTGAGGTGG 150
CahMYB11 CTGCAAGAACAGGACTCAATAGGTGCAGAAAAAGTTGCAGGTTGAGGTGG 150
 ClMYB11 CCGCTAGAACAGGGCTGAATAGGTGCAGAAAAAGTTGCAGATTGAGGTGG 129

CgsMYB11 CTCAACTATCTGAAGCCGGGCATAAACCGTAGAGAGTTTCAAGAAGATGA 200
CgaMYB11 CTCAACTATCTGAAGCCGGGCATAAACCGTAGAGAGCTTCAAGAAGATGA 200
CahMYB11 CTCAACTATCTGAAGCCGGGCATAAACCGTAGAGAGCTTCAAGAAGATGA 200
 ClMYB11 CTCAACTATCTTAAACCGGGCATAAACCGTAAAGAGCTTCAAGAAGATGA 179

CgsMYB11 AGTTGACTTGATCATCAGGCTTCATAAGCTTCTTGGCAATAGATGGTCAC 250
CgaMYB11 AGTTGACTTGGTCATCAGGCTTCATAAGCTTCTTGGCAATAGATGGTCAC 250
CahMYB11 AGTTGACTTGATCATCAGGCTTCATAAGCTTCTTGGCAATAGATGGTCAC 250
 ClMYB11 AGTTGACTTGATCATCAGGCTTCATAAGCTTCTTGGAAATAGATGGTCAC 229

CgsMYB11 TTATTGCAGGAAGGCTACCGGGAAGAACATCCACCCAAGTAAAGAATTAC 300
CgaMYB11 TTATTGCGGGAAGGCTTCCGGGAAGAACATCCACCCAAGTAAAGAATTAC 300
CahMYB11 TTATTGCAGGAAGGCTTCCGGGAAGAACATCCACCCAAGTAAAGAATTAC 300
 ClMYB11 TTATTGCAGGAAGACTTCCCGGAAGAACATCCACCCACGTAAAGAATTAC 279

CgsMYB11 TGGAATGCCCATATAGCTAAAAAGTGGAGATCGTCATCCAAAGCTGCACC 350
CgaMYB11 TGGAATGCCCGTACAGCTAAAAAGTGGAGATCGTCATCCAAATCTGCAGC 350
CahMYB11 TGGAATGCCCATATAGCTAAAAAGTGGAGATCGTCATCCAAAGCTGCAGC 350
 ClMYB11 TGGAATGCCCATATAGCTAAAAAGTGGAGATCGTCATCAAA------AGT 323

CgsMYB11 TGCAGAATCAAAATCATCATCTTATAAAGGAA---AACAGCAAGAATATT 397
CgaMYB11 TGCAGAATCAAAATCATCATC---TAAAGGAA---AACAACAACAATATT 394
CahMYB11 TGCAGAATCAAAATCATCATC---TAAAGGAA---AACAGCAAGAATATT 394
 ClMYB11 TGCAGAATCAAAATCATCATC---AAAAGGAAAGCAGCAGCAAAAATATC 370

CgsMYB11 CAGTAAACGTCATAAAGCCTATTGCTAGAAGAGCTCCCAAAATGATCGAT 447
CgaMYB11 CAGTAAACGTTATAAGGCCTATTGCTAGAAGAGCTCCCAAAATGATCGAT 444
CahMYB11 CAGTAAACGTCATAAAGCCTATTGCTAGAAGAGCTCCCAAAATGATCGAT 444
 ClMYB11 CAGTCAACGTCATAAGGCCTATTGCTAGAAGAGCTCCCAAAATGATCAAG 420

CgsMYB11 TTTGGTATGACCATGAATAACAATAATATATGCAGCATGTCCACCTCTCA 497
CgaMYB11 TTTGGTATGATCATGAATAACAATAATATATGCAGCAGGTCCACCTCTCA 494
CahMYB11 TTTGGTATGATCATGAATAACAATAATATATGCAGCATGTCCACCTCTCA 494
 ClMYB11 TTTGGT------ATGAATGATAATAATAAATGCAGCAGGTCCACCTCTCA 464

CgsMYB11 GCTGCCGC---------CTCATCTAGATTGTACCGGAATAATAAATACAG 538
CgaMYB11 GCTGCCGC---------CTCATCTAGATTGTACCGAAATAATAAATACAG 535
CahMYB11 GCTGCCGC---------CTCATCTAGATTGTACCGAAATAATAAATACAG 535
 ClMYB11 GCCGCCGCCGCCTCCATCTCATCTTGATTGTAACGAAATAATAAATGAAG 514
```

Fig. S1. (to be continued on the next page)

```
CgsMYB11  ACAAGGAGGTGATGAATTGGTTGCAGAGATTATTGGACGATGATGATTTA  588
CgaMYB11  ACAAGGAGGTGATGAATTGGTTGGAGAGATTATTGGACGATGATGATTTA  585
CahMYB11  ACAAGGAGGTGATGAATTGGTTGCAGAGATTATTGGACGATGATGATTTA  585
 ClMYB11  ACAACGAGGTGATGAATTGGTTGGAGAGGTTATTGGACGATGATGATTTA  564

CgsMYB11  ATTGGTTTTGGAGGAGACGGTGGCTCTGCCGCCTCAGAAGGCCACTGTAC  638
CgaMYB11  ATTGGTGGTGGAGGAGACGGTGGCTCTGCCGCCTCAGAAGGCCACTGTAC  635
CahMYB11  ATTGGTGGTGGAGGAGACGGTGGCTCTGCCGCCTCAGAAGGCCACTGTAC  635
 ClMYB11  ATTGGTGGAGGAGGAGACAGTGGCTGTGCCGCCTCAGAAGGCCACTGTAT  614

CgsMYB11  CACCGTAGGTGGTGGGTATATGTCGACTCCATGA                  672
CgaMYB11  CACCGTAGGTGGTGGGTATATGTCGACTCCATGA                  669
CahMYB11  CACCGTAGGTGGTGGGTACATGTCGACTCCATGA                  669
 ClMYB11  CGTCGTAGAAGGTGGGTATATATCGACTGCATGA                  648
```

Fig. S1. (to be continued on the next page)

D

```
 CgsMYB12  ATGAAGGAAGGTCTAAGGAAGGGTGCTTGGAGTGCAGAAGAAGATGCTCT  50
CgsMYB12W  ATGAAGGAAGGTCTAAGGAAGGGTGCTTGGAGTGCAGAAGAAGATGCTCT  50
 CahMYB12  --------------------GGGTGCTTGGAGTGCAGAAGAAGATGCTCT  30
  ClMYB12  ATGAACGGAGGTCTAAGGAAGGGTGCTTGGAGTGAAGAAGAAGATGCTCT  50

 CgsMYB12  CCTCAAGCAATGTATTCAAATTTATGGAGAAGGCAAATGGCATCTTGTTC  100
CgsMYB12W  CCTCAAGCAATGTATTCAAATTTATGGAGAAGGCAAATGGCATCTTGTTC  100
 CahMYB12  CCTCAAGCAATGTATTCAAATTTATGGAGAAGGCAAATGGCATATTGTTC  80
  ClMYB12  ACTCAGGCAATGCATTCAAACTTATGGAGAAGGCAAATGGCATCTTGTTC  100

 CgsMYB12  CCGCCAGAGCAGGGCTAAATAGGTGTAGAAAAGGTTGCAGATTGAGGTGG  150
CgsMYB12W  CCGCCAGAGCAGGGCTAAATAGGTGTAGAAAAGGTTGCAGATTGAGGTGG  150
 CahMYB12  CCGCCAGAGCAGGGCTAAATAGGTGTAGAAAAGGTTGCAGATTGAGGTGG  130
  ClMYB12  CCGCTAGATCAGGGCTGAATAGGTGCAGAAAAGGTTGCAGGTTGAGGTGG  150

 CgsMYB12  CTCAACTATCTGAAGCCAGGCATAAACCTAAAAGAGCTTCAAGATGATGA  200
CgsMYB12W  CTCAACTATCTGAAGCCAGGCATAAACCTAAAAGAGCTTCAAGATGATGA  200
 CahMYB12  CTCAACTATCTGAAGCCAGGCATAAACCTAAAAGAGCTTCAAGATGATGA  180
  ClMYB12  CTCAACTATTTGAAGCCCGGCATAAACCTAACAGAGCTTCAACATGATGA  200

 CgsMYB12  AGTTGACTTGATCCTCAAACTTCACAAGCTTCTTGGCAACAAATGGTCAC  250
CgsMYB12W  AGTTGACTTGATCCTCAAACTTCACAAGCTTCTTGGCAACAAATGGTCAC  250
 CahMYB12  AGTTGACTTGATCCTCAAACTTCACAAGCTTCTTGGCAACAAATGGTCAC  230
  ClMYB12  ACTTGACTTGATCTTCAAACTTCACAAGCTTCTTGGCAACAAATGGTCAC  250

 CgsMYB12  TTATAGCAGGAAGACTTCCGGGAAGAACATGCAATTATATAAAGAATTAC  300
CgsMYB12W  TTATAGCAGGAAGACTTCCGGGAAGAACATGCAATTATATAAAGAATTAC  300
 CahMYB12  TTATAGCAGGAAGACTTCCAGGAAGAACATGCAATTATATAAAGAATTAC  280
  ClMYB12  TTATTGCAGGAAGACTTCCCGGAAGAACATGCAACTACATAAAGAATTAC  300

 CgsMYB12  TGGAACTCCAATATTGCTGCTAAAAAGTGGAAATCAAGAGAAAAGCAGCA  350
CgsMYB12W  TGGAACTCCAATATTGCTGCTAAAAAGTGGAAATCAAGAGAAAAG-AGCA  349
 CahMYB12  TGGAACTCCAATATTGCTGCTAAAAAGTGGAAATCAAGAGAAAAGCAGCA  330
  ClMYB12  TGGAACTCCCATTTTACTGCTAAAAAGTGTAAATCAAGAAAAAAGCTGAA  350

 CgsMYB12  AGATTCGGTCAAAGTTAAAGCCATAAGGCCTATTGTACGAAGAGCTCCCA  400
CgsMYB12W  AGATTCGGTCAAAGTTAAAGCCATAAGGCCTATTGTACGAAGAGCTCCCA  399
 CahMYB12  AGATTCGGTCAAAGTCAAAGCCATAAGGCCTATTGTACGAAGAGCTCCCA  380
  ClMYB12  CGATTCGGTCAAAGT------CATAAGGCCTATTGTACGAAGAGCTCATA  394

 CgsMYB12  AAATAATCAACTTTGGAATGAATAATAATAAT-------ATA--GGAACC  441
CgsMYB12W  AAATGATCAACTTTGGAATGAATAATAATAAT-------ATA--GGAACC  440
 CahMYB12  AAATGATCAACTTTGGAATGAATAATAATAATAATAACAATAATGGAAGC  430
  ClMYB12  AAATGATCAAGTTCGGTAATAATAATAATAATAATAAC-ATA--GGGACC  441

 CgsMYB12  ACCTCTCA---------------------------GCCTCATTGTAACGG  464
CgsMYB12W  ACCTCTCA---------------------------GCCTCATTGTAACGG  463
 CahMYB12  ACCTCTCA---------------------------GCCTCATTGTAACGG  453
  ClMYB12  ACCTCTCGGCCGCCGTCACTGCCGCCAACACCTCCGCCTCATTGTAACGG  491

 CgsMYB12  AATAACTAGGGACGACGAGGTGATGAATTGGTTGGATAGGTTACTAATGG  514
CgsMYB12W  AATAACTTGGGACGACGAGGTGATGAATTGGTTGGATAGGTTACTAATGG  513
 CahMYB12  AATAACTACAGACGACGAGGTGATGAATTGGTTGGATAGGTTACTAATGG  503
  ClMYB12  AATAAATACAGACAACGAGGTGGTGAATTGGTTGGATAGGTTACTAGTGG  541
```

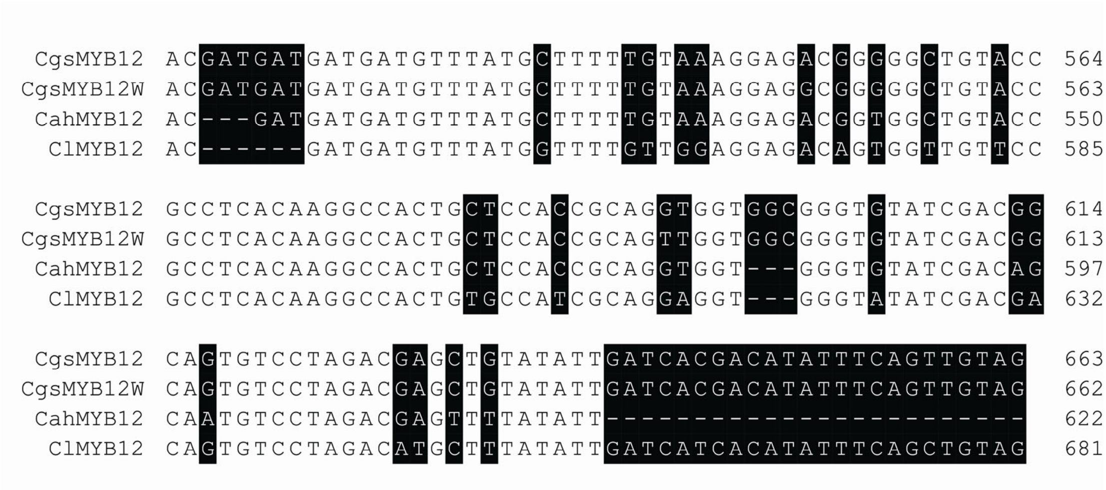

Fig. S1. Alignment of *Clarkia R2R3-MYB* sequences (*A*) *MYB1*, (*B*) *MYB6*, (*C*) *MYB11* and (*D*) *MYB12* from *C. gracilis sonomensis*, *C. g. albicaulis*, *C. amoena huntiana* and *C. lassenensis*. If the nucleotides at each column are not identical, this column is highlighted in a black background. The sequences retrieved from GenBank are: *CgsMYB1* (KX592432), *CgaMYB1* (KX592431), *ClMYB1* (KX592428), *CgsMYB6* (MT425534), *CgsMYB11* (MT425536), *CgsMYB12* (MT425538) and *CgsMYB12*^{W} (MT425537). Other sequences were generated in this study and were deposited in GenBank under the accession numbers MT796894-MT796902. *CgsMYB12*^{W} is the nonfunctional copy of *CgsMYB12*, with the presence of a deletion (highlighted in a gray background) that creates a premature stop codon (highlighted in a red background). The conserved MYB DNA-binding domain, consisting of R2 and R3 repeats, is indicated in pink and blue letters. The fragment corresponding to the conserved amino acid motif specific to the anthocyanin-regulating *R2R3MYB*s is indicated in orange letters.

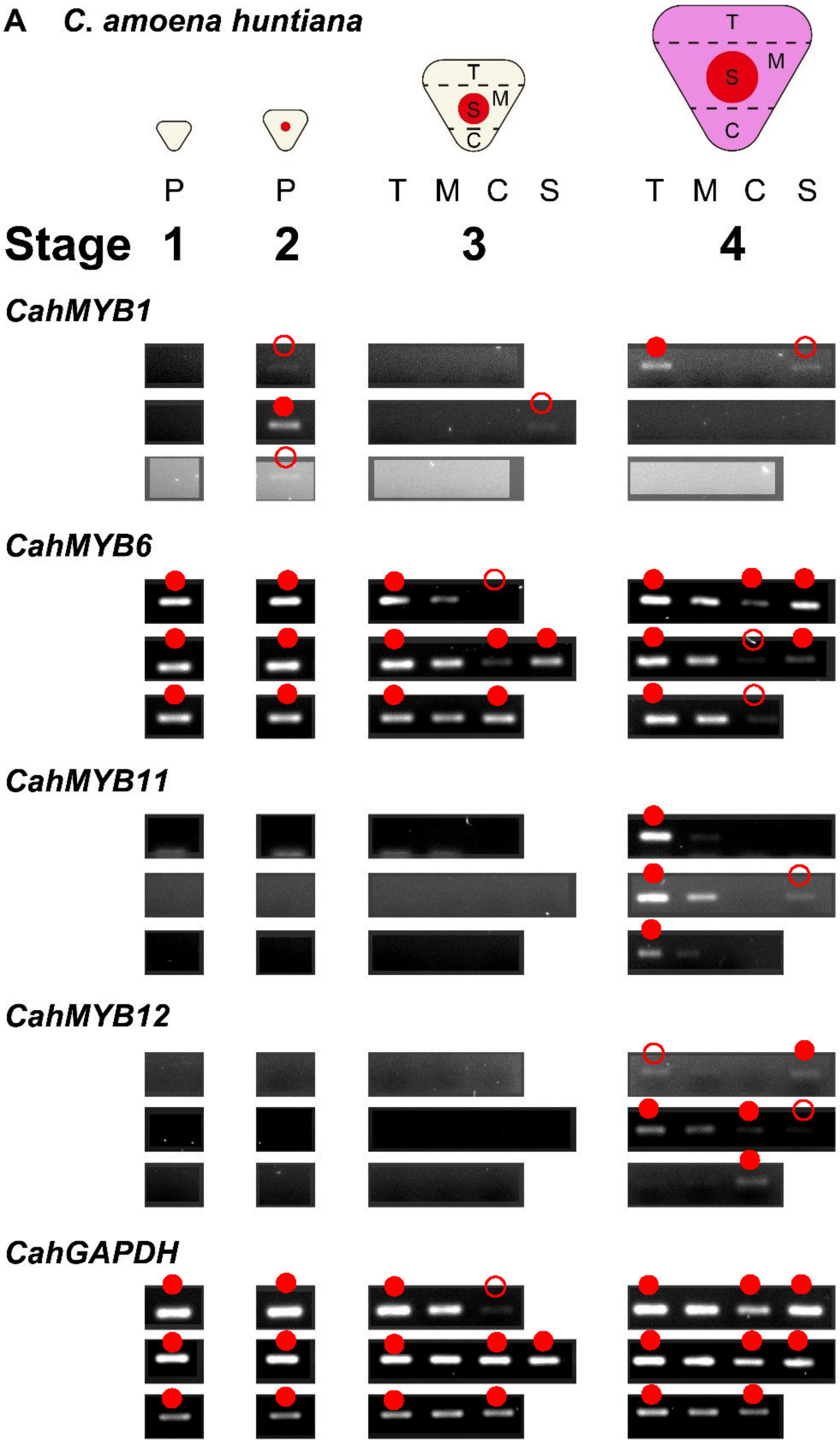

Fig. S2. (to be continued on the next page)

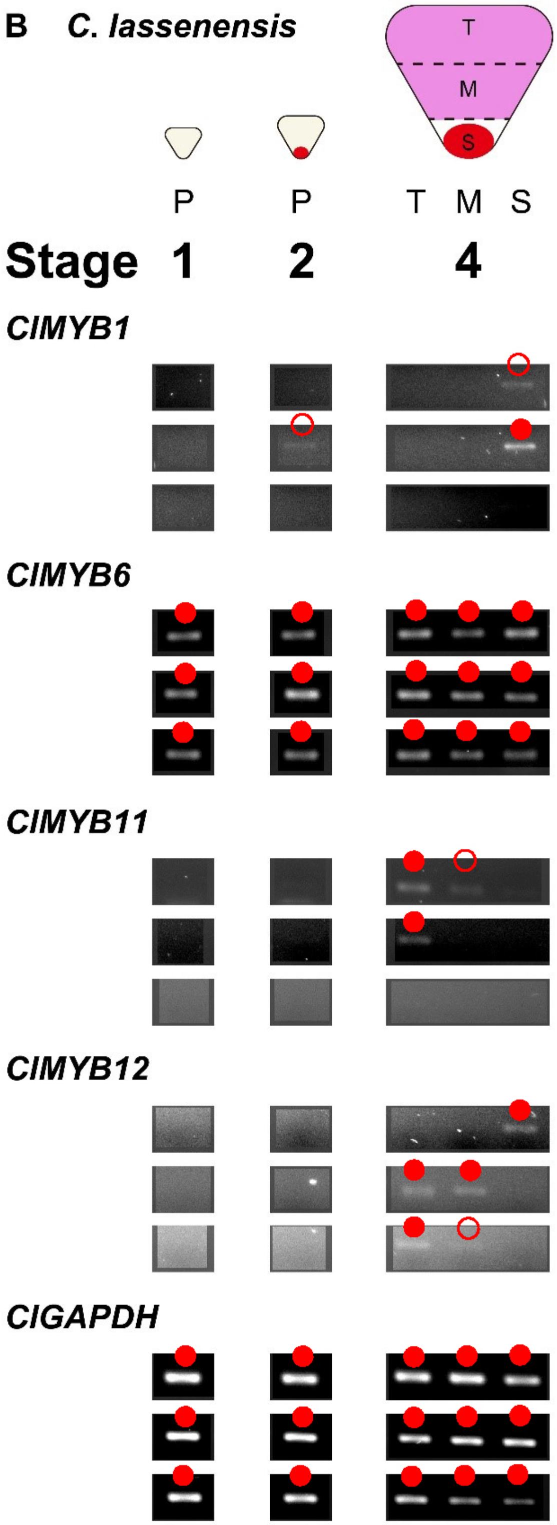

Fig. S2. (to be continued on the next page)

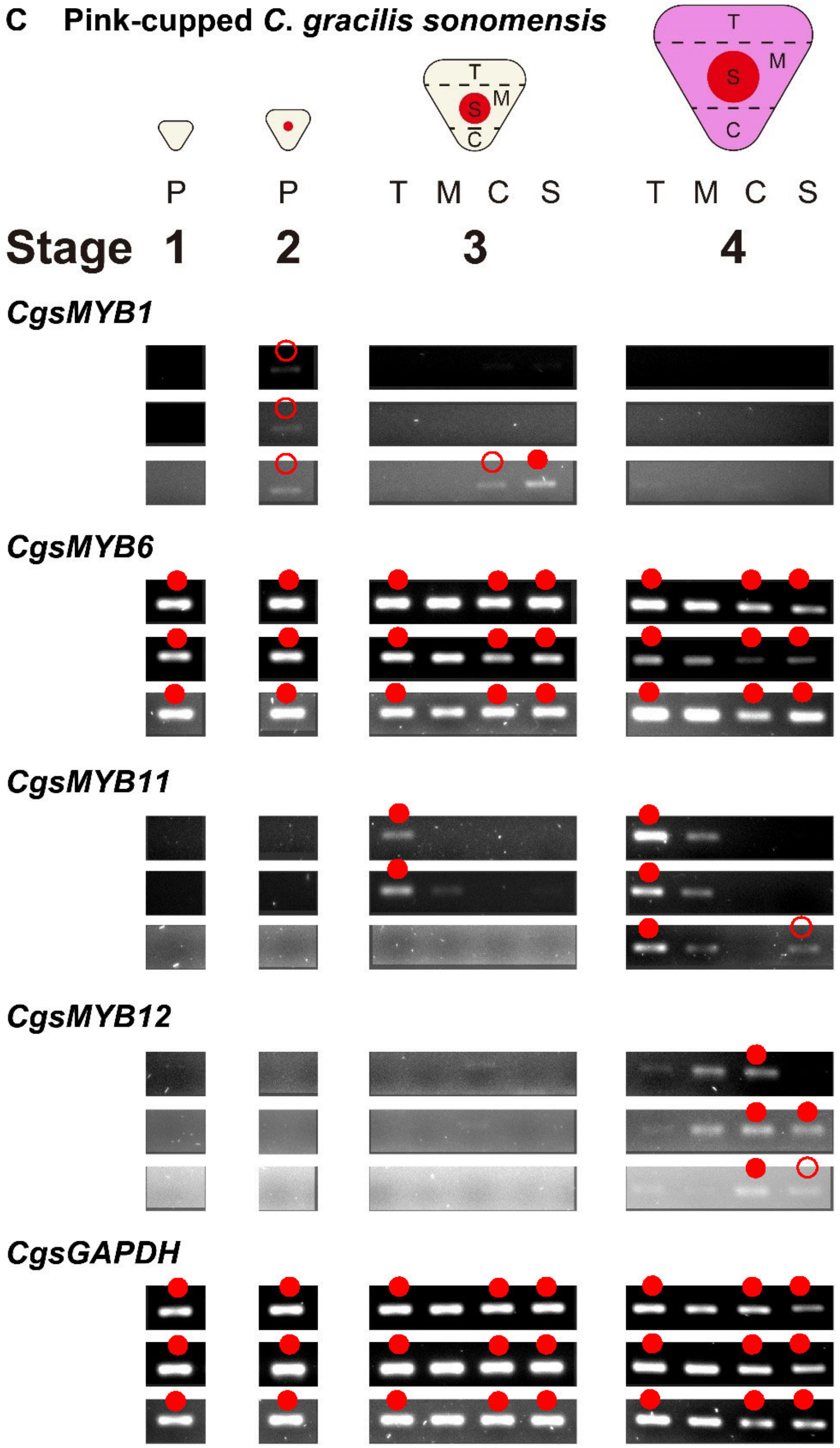

Fig. S2. (to be continued on the next page)

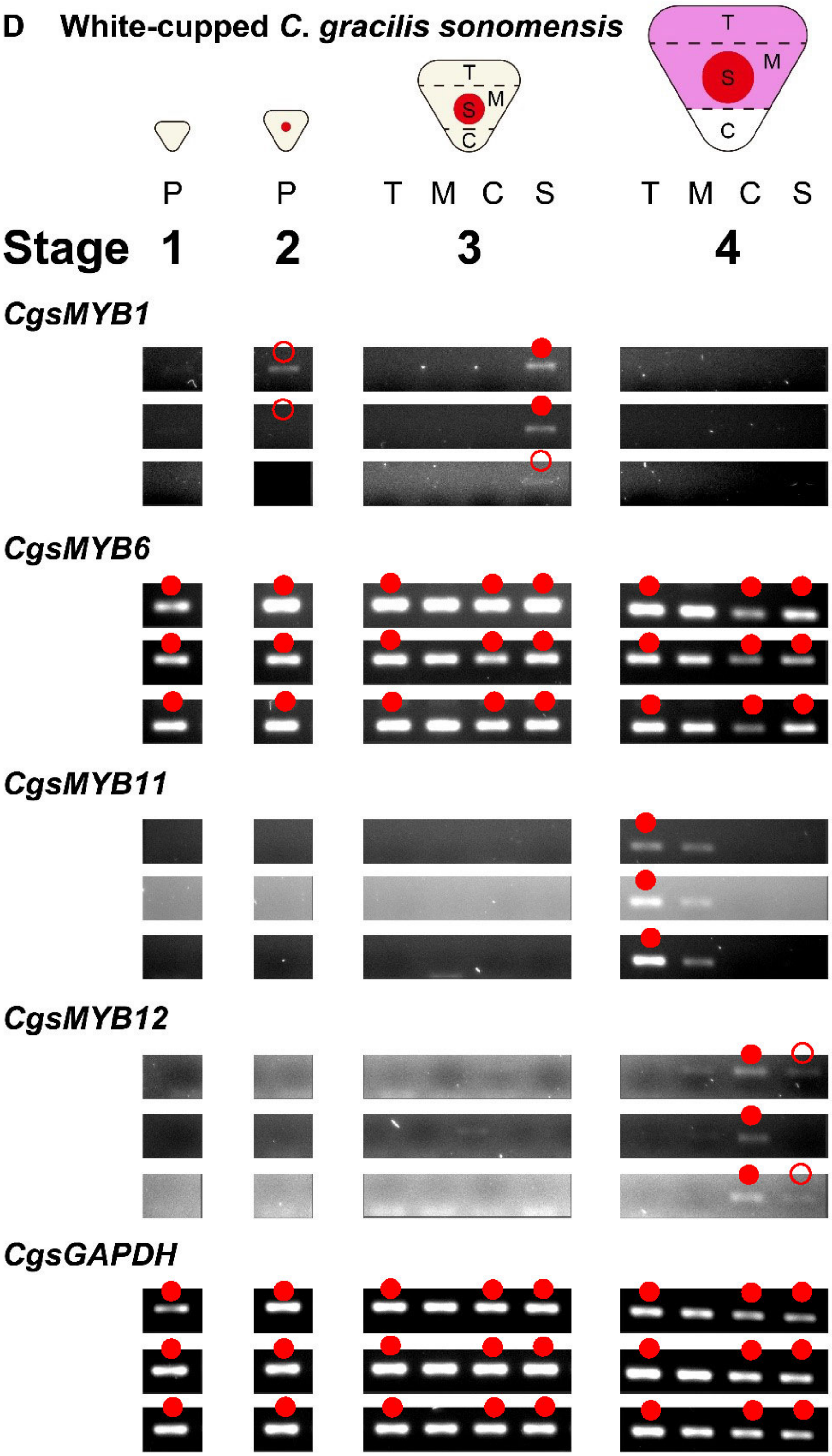

Fig. S2. (to be continued on the next page)

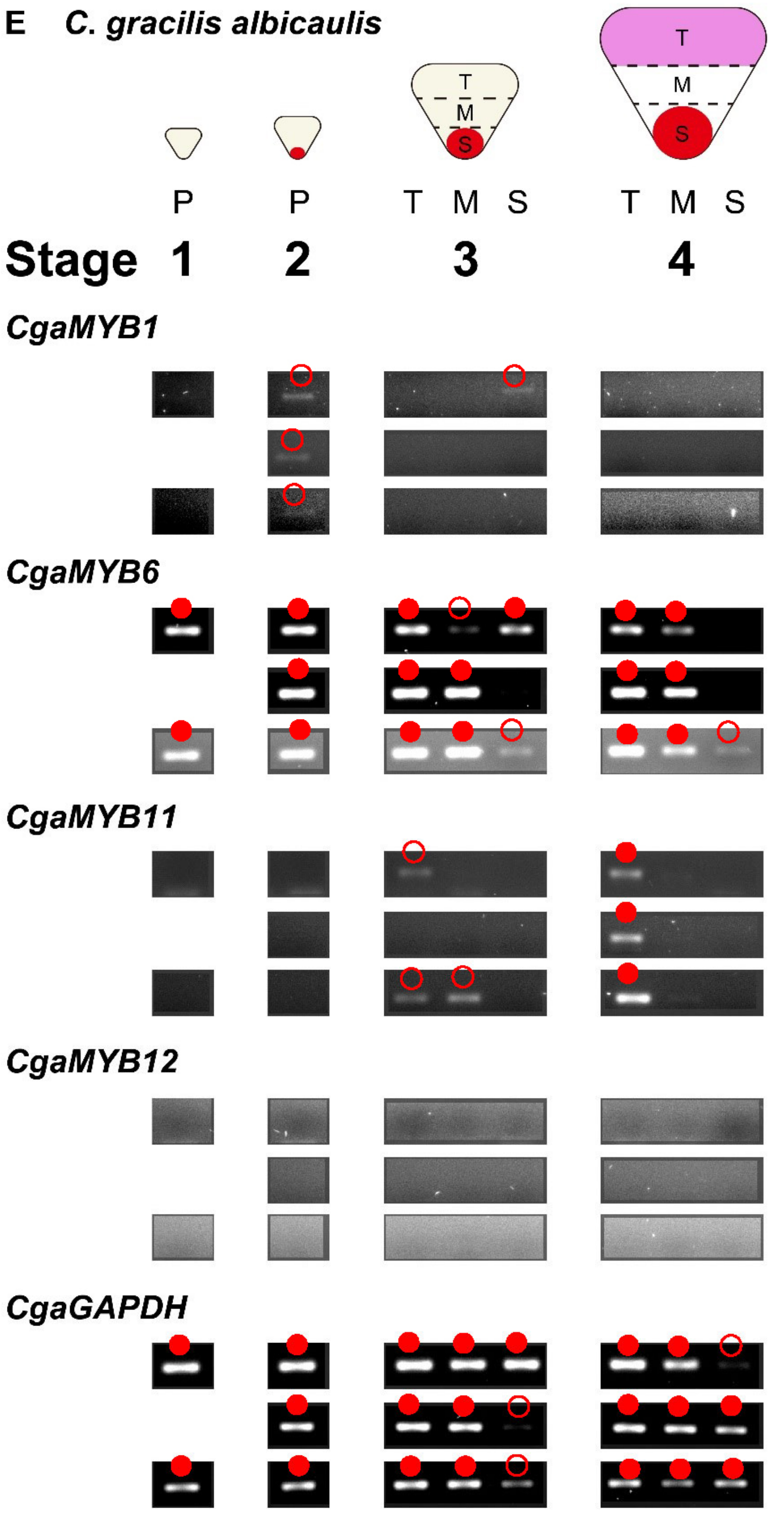

Fig. S2. Spatiotemporal expression of *MYB1*, *MYB6*, *MYB11* and *MYB12* in the petals of (*A*) *Clarkia amoena huntiana*, (*B*) *C. lassenensis*, (*C*) pink-cupped *C. gracilis sonomensis*, (*D*)

white-cupped *C. g. sonomensis*, (*E*) *C. g. albicaulis*. Flower buds were collected from three plants from each (sub)species/phenotypes. For Stages 1 and 2, the whole petals (P) were used. For Stages 3 and 4, the petals were dissected into sections according to coloration: T (top), M (middle, excluding the spot), C (cup) and S (spot). For *C. lassenensis*, Stages 2 and 3 were combined into Stage 2 due to a small petal size. A constitutively expressed gene *GAPDH* was included for cDNA quality control. Red circles indicate detected bands. Filled circles correspond to “+” and open circles to “(+)” in fig. 4.

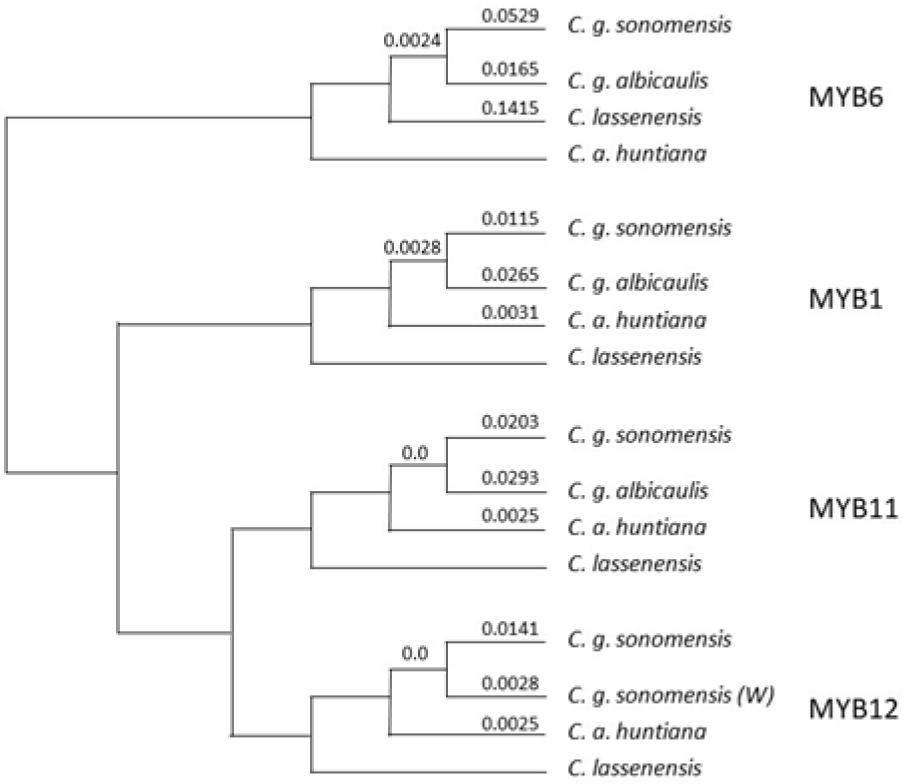

Fig. S3. Gene tree used to estimate d_S (synonymous substitution rate) along branches. Numbers above branches indicated estimated d_S. (W) indicates the nonfunctional allele of *MYB12* in *Clarkia gracilis sonomensis*.

Methods S1. Transcriptomics

To perform a comprehensive survey on which transcripts were expressed differentially between the pink background (the most distal portion) and the white band (the central portion) of the *Clarkia gracilis albicaulis* petals, we sequenced RNA extracted from these two petal regions. The petal regions were collected by dissecting flower buds (approximately 1 day before flowering) from four plants. Total RNA of pink background and white band was individually extracted using Spectrum Plant Total RNA Kit (Sigma-Aldrich, St. Louis, MO, USA). RNA samples of the two petal regions for library construction were prepared by pooling equal amounts of RNA from each plant. Prior to library construction, RNA quality was examined using Bioanalyzer Agilent RNA 6000 Nano Kit (Agilent Technologies, Santa Clara, CA, USA). We followed the protocol described in Supporting Information Methods S2 in Lin and Rausher (2021) to construct, barcode and sequence the libraries.

Bioinformatic analyses, including transcriptome assembling, identification of candidate genes for anthocyanin production and estimation of gene expression, were conducted as described in Lin and Rausher (2021).

Raw sequence reads generated in this study were deposited under NCBI Bioproject PRJNA721169 (Sequence Read Archive accessions: SRR14226355 and SRR14226356). The assembled transcriptomes were deposited at the NCBI Transcriptome Shotgun Assembly under the accessions GJDS00000000 and GJDT00000000. FPKM data are available at http://doi.org/10.5281/zenodo.4699974.

References cited in Supplementary Material

Lin R-C, Rausher MD. 2021. *R2R3-MYB* genes control petal pigmentation patterning in *Clarkia gracilis* ssp. *sonomensis* (Onagraceae). *New Phytol.* 229(2):1147–1162.

Martins TR, Jiang P, Rausher MD. 2017. How petals change their spots: *cis*-regulatory re-wiring in *Clarkia* (Onagraceae). *New Phytol.* 216(2):510–518.
